# P-1450. Impact of Multi-morbidities on Quality of Life Among People Living with HIV at a tertiary care center in India: A cross-sectional study

**DOI:** 10.1093/ofid/ofae631.1622

**Published:** 2025-01-29

**Authors:** Sai Prasad Ramachandran, Abhigna Manam

**Affiliations:** Armed Forces Medical College, Pune, India, Thrissur, Kerala, India; Armed Forces Medical College, Pune, India, Thrissur, Kerala, India

## Abstract

**Background:**

India is home to an estimated 2.4 million People Living with HIV (PLHIV). The term "multi-morbidity" refers to the simultaneous presence of 2 or more chronic medical conditions in an individual, the prevalence of which is rising. The aim of our study is to assess the impact of multi-morbidities on Quality of Life (QoL) in PLHIV attending the Antiretroviral Therapy (ART) center at a tertiary care hospital in Western Maharashtra, India.

**Figure:**
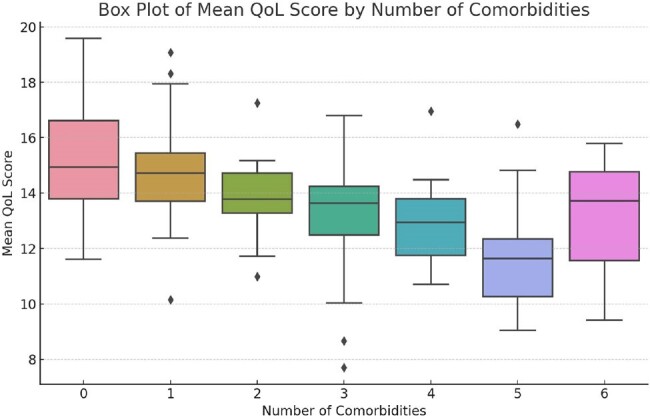

**Methods:**

This was a cross-sectional study, over 2 months with 285 patients recruited. After ethical clearance was obtained, patients were interviewed to gather comprehensive sociodemographic history, with information on treatment and comorbidities obtained from patient charts. The QoL was measured using the WHOQOL-HIV BREF questionnaire which evaluates QoL across six domains: Physical, Psychological, Independence, Social, Environment, and Spiritual.

**Results:**

The cohort exhibited an average of 2.2 comorbidities (SD = 1.4), with a notable prevalence of cardiovascular diseases and diabetes. Participants with 3 or more comorbidities demonstrated significantly lower QoL scores in the Physical (mean = 10.4, SD = 1.8, p < 0.01) and Psychological (mean = 11.9, SD = 1.5, p < 0.05) domains compared to those with fewer comorbidities. A longer duration of ART correlated positively with higher scores in the Independence domain (r = 0.26, p < 0.05). Elevated CD4 counts were linked with improvements in the Environmental and Psychological health domains. (p < 0.05) Participants from higher socioeconomic backgrounds reported significantly better scores in the Environmental and Spiritual domains (p < 0.05). Younger patients (< 40 years) reported better Social and Spiritual QoL scores compared to older individuals Gender-specific analysis indicated that females reported higher scores in the Social and Environmental domains. (p < 0.01).

**Conclusion:**

There is a pressing need for effective management strategies that encompass comprehensive care approaches to address the complex health needs of this population. Enhancing routine screening for comorbidities and educating patients about the implications of multi-morbidities can empower PLHIV to improve QoL.

**Disclosures:**

**All Authors**: No reported disclosures

